# Supplementary material for: Bioengineering of Genetically Encoded Gene Promoter Repressed by the Flavonoid Apigenin for Constructing Intracellular Sensor for Molecular Events
Source: Biosensors (Basel). 2021 Apr 28;11(5):137. doi: 10.3390/bios11050137 (PMC8147076; doi:10.3390/bios11050137)
Supplement: Supplementary file 1 [file biosensors-11-00137-s001.zip › biosensors-1132903-supplementary.pdf]

biosensors

MDPI

Supplementary information

Bioengineering of Genetically Encoded Gene Promoter Repressed by the Flavonoid Apigenin for Constructing Intracellular Sensor for Molecular Events

Nicole M. Desmet [1,2], Kalyani Dhusia [1], Wenjie Qi [1], Andrea I. Doseff [3,4], Sudin Bhattacharya [1,4] and Assaf A. Gilad [1,2,5,*]

[1] Department of Biomedical Engineering, Michigan State University, East Lansing, MI 48824, USA; nicole.m.desmet.gr@dartmouth.edu (N.M.D.); kalyani.dhusia@einsteinmed.org (K.D.); qiwenjie@msu.edu (W.Q.); sbhattac@msu.edu (S.B.)
[2] Division of Synthetic Biology and Regenerative Medicine, Institute for Quantitative Health Science and Engineering, Michigan State University, East Lansing, MI 48824, USA
[3] Department of Physiology, Michigan State University, East Lansing, MI 48824, USA; doseffan@msu.edu
[4] Department of Pharmacology and Toxicology, Michigan State University, East Lansing, MI 48824, USA
[5] Department of Radiology, Michigan State University, East Lansing, MI 48824, USA
* Correspondence: gilad@msu.edu

HeLa cells were chosen versus HEK293FT cells because HeLa cells have stronger adhesive properties and therefore better handled the wash steps. To enhance efficiency, each trial was done in a 96-well microplate. Transfection protocol optimization consisted of varying cell concentration, transfection reagent concentrations, DNA concentration, and ratio to best enhance transfection efficiency. To determine the most efficient conditions, cells were transfected with pcDNA3.1/V5-His-TOPO/lacZ since the CYP1A1 plasmid is of similar size, and LacZ levels were quantified using a beta-galactosidase expression kit and microplate reader. Next, treatment conditions were optimized. In a time-lapse experiment, 48 hours was found to be the optimal treatment duration.

To simulate the ligand-receptor binding, the AHR/ARNT complex was used, shown in Figure S1.

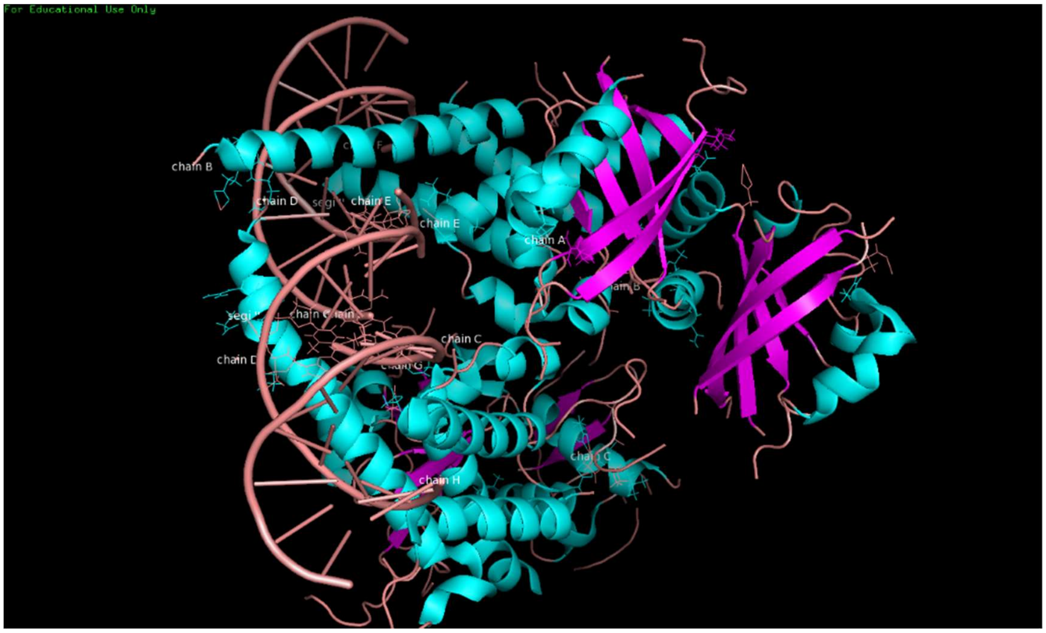

Figure S1. AHR/ARNT complex modeled by UCSF Chimera Viewer.
